# Overexpression of SGLT2 in the kidney of a *P. gingivalis* LPS-induced diabetic nephropathy mouse model

**DOI:** 10.1186/s12882-021-02506-8

**Published:** 2021-08-23

**Authors:** Koichiro Kajiwara, Yoshihiko Sawa

**Affiliations:** 1 Department of Oral Growth & Development, Fukuoka Dental College, 2-15-1 Tamura, Sawara-ku, Fukuoka, 814-0193 Japan; 2 Department of Oral Function & Anatomy, Okayama University Graduate School of Medicine, Dentistry and Pharmaceutical Sciences, 2-5-1 Shikata-cho, Okayama, Kita-ku 700-0914 Japan

**Keywords:** *P. gingivalis*, LPS, Diabetic nephropathy, SGLT2

## Abstract

**Background:**

The overexpression of sodium-glucose cotransporter 2 (SGLT2) in diabetic kidneys has been reported. It has also been established that the diabetic glomerular endothelium expresses the toll-like receptors TLR2 and TLR4. The present study aims to examine the renal SGLT2 induction by the TLR2/4 ligand *Porphyromonas* (*P.*) *gingivalis* lipopolysaccharide (Pg-LPS) in mouse diabetic nephropathy.

**Methods:**

Immunohistochemical study and tissue RT-PCR analyses were performed on mouse kidneys in streptozotocin (STZ)-induced diabetic ICR mice (STZ-ICR), in healthy ICR mice administered Pg-LPS (LPS-ICR), and in diabetic ICR mouse kidneys with Pg-LPS-induced nephropathy (LPS-STZ).

**Results:**

In the quantitative analysis of blood sugar levels, the mean time to reach 600 mg/dl was shorter in the LPS-STZ than in the STZ-ICR kidneys. The rise in blood glucose levels was significantly steeper in the LPS-STZ than in the STZ-ICR kidneys. According to these data the LPS-STZ model suggests a marked glucose intolerance. The expression of SGLT2 was significantly stronger in the whole of the renal parenchyma of the LPS-STZ than in the LPS-ICR or in the STZ-ICR. The expression of SGLT2 was observed both in the renal tubules and around the renal tubules, and in the glomeruli of the LPS-STZ kidneys. In the analysis by tissue real-time PCR and cell ELISA, the expression of the SGLT2 gene and protein was significantly stronger in the LPS-STZ than in the LPS-ICR or in the STZ-ICR. There were no differences in the renal SGLT2 production in the LPS-ICR and the STZ-ICR kidneys.

**Conclusions:**

Abnormally high renal expression of SGLT2 occurs in diabetic kidneys with *P. gingivalis* LPS. Periodontitis may be an exacerbating factor in diabetic nephropathy as well as in diabetes.

## Background

The pathological mechanisms which cause individual differences in the progression of diabetes and in the accompanying complications are not fully elucidated. Diabetic glomerulosclerosis exhibits characteristics such as: diffuse lesions due to increased mesangial matrix including type I collagen by the production of several different cytokines through the recognition of advanced glycation end products (AGE) in a hyperglycemic environment [1]; nodular lesions with circular enlargement of the mesangial region as Kimmelstiel-Wilson nodules; exudative lesions with a fibrin cap in which plasma components are stored in the hemispheric space between glomerular tuft endothelial cells and the basement membrane and in a capsular drop filled with plasma components stored between the Bowman’s epithelium and the basement membrane [2]. It has been shown that hyperglycemia induces the TLR2/4 expression via PKC-α/δ with NADPH oxidase activation in leukocytes [3, 4]. The metabolic recognition of AGE by the renal toll-like receptor (TLR) like the AGE receptor has been suggested as one candidate for the occurrence of diabetic nephropathy [5–7]. The TLR recognizes pathogen-associated molecular patterns like lipopolysaccharide (LPS) in microorganism components, and elevated levels of TLR2/4 expression have been reported in leukocytes in diabetic patients [8–12]. The renal TLR ligand binding induces the production of inflammatory cytokines which activate inflammatory events in kidneys [13–17]. The periodontal pathogen *Porphyromonas* (*P.*) *gingivalis* LPS (Pg-LPS) induces the production of inflammatory agents by host defense systems recognizing lipid A and co-existing molecules of TLR2 and TLR4 [18–20]. The Pg-LPS acts as a periodontal pathogen leading to periodontal tissue destruction and is also a risk factor in cardiovascular disorders [21, 22]. It is thought that patients with severe periodontitis are liable to cause bacteremia of the systemic circulation with oral microorganisms, resulting in the renal accumulation of bacterial components or the immune complexes like in IgA nephropathy [23–25]. Intestinal microorganisms also enter the circulation but are directly sterilized in the liver while oral microorganisms enter the systemic circulation directly; this has led to the suggestion that head and neck infections spread throughout the body and enter the kidney via the systemic circulation [24–27].

We recently reported that the glomerular endothelium expresses TLR2 and TLR4 in streptozotocin (STZ)-induced diabetic mice [28], and that all Pg-LPS-administered diabetic mice reached humane endpoints within the survival time of all diabetic mice and Pg-LPS-administered non-diabetic mice [29]. The diabetic mice administered Pg-LPS showed nephropathy with glomerulosclerosis based on the glomerular accumulation of type 1 collagen and inflammatory cytokines. The Pg-LPS administrated diabetic mice showed high levels of sugar/protein in the urine and blood urea nitrogen (BUN)/creatinine (CRE). The progress of these effects was suppressed by the administration of the TLR4 inhibitor Eritoran [30]. This has led to suggestion that periodontitis may be a risk factor in exacerbating nephropathy in diabetic patients [28–30]. It has been reported that there is overexpression of sodium-glucose cotransporter 2 (SGLT2) in diabetic kidneys [31–33]. The SGLT actively transport glucose with the sodium transport across the intestinal epithelial cells and the proximal renal tubule epithelial cells against the concentration gradient. Approximately 90% of the filtered renal glucose is reabsorbed at the early convoluted segment of the proximal tubule by SGLT2. The glucose transported into proximal tubular cells exits the basolateral cell border and are returned to the circulation [32, 33]. Recently, SGLT2 inhibitors are used to suppress diabetic nephropathy [32–34], however, the mechanisms that cause the abnormal expression of SGLT2 are still not fully elucidated. With the increased expression of SGLT2 in diabetic patients, SGLT2 has been suggested as an important factor in exacerbating diabetes in a production amount-dependent manner. The mechanism of the increase in the renal glucose reabsorption for hyperglycemia involves the overexpression of glucose transporter genes in the proximal tubule [31–33]. In both rodent diabetic models [35–37] and humans [31] the upregulation of SGLT2 gene/protein has been reported in the renal proximal tubular cells. In cultured proximal tubular cells isolated from type II diabetic patients and healthy controls SGLT2 and GLUT2 mRNA levels and glucose transport are significantly higher in the diabetes group [31], and diabetic rodent models show similar results. It has been thought that increased plasma glucose ultimately leads to increased SGLT2 and GLUT2 mRNA/protein expression in the renal proximal tubular cells [32, 33]. Considering the possibility of periodontitis as a risk factor for exacerbating nephropathy, it may be postulated that the SGLT2 overexpression could be provoked by *P. gingivalis* in diabetic kidneys. The present study aims to examine the expression of SGLT2 in mouse kidneys with Pg-LPS-induced diabetic nephropathy.

## Methods

### Animals

All processes for the upkeep of animals and experiments were conducted in the Fukuoka Dental College Animal Center following the conditions and procedures described elsewhere [30]. This study was conducted to investigate the SGLT2 expression in the Pg-LPS-induced diabetic nephropathy model mice. The protocol of the animal investigation was approved by the Animal Experiment Committee of Fukuoka Dental College (No. 19010). The 4-week-old male mice of the ICR closed line were purchased from a commercial vendor (Kyudo, Fukuoka, Japan). The animal number was decided according to rules by the Animal Experiment Committee of Fukuoka Dental College based on the appropriate number of animals in biomedical research from the viewpoint of animal welfare [38]. In the case of genetically identical animal groups of the same lineage, 5 or more animals per group, and the test using 4 groups including the control group, are considered appropriate. This study used 4 groups (8 mice in each group): non-treated control (data not shown), LPS-administered non-diabetic control, diabetic control, and Pg-LPS-administered diabetic experimental. All specimens were collected from euthanized mice according to the ARRIVE guidelines. The humane endpoints were assessed daily and mice reaching humane endpoints were euthanized by induction anesthesia with intraperitoneal injections of sodium pentobarbital and cervical dislocation. In this study, data for all the mice were included in the experimental and control data, and there were no exclusions. Recently, we have established the Pg-LPS-injected diabetic nephropathy mouse model as described elsewhere [30]. Briefly, a single intraperitoneal injection of STZ (Sigma-Aldrich Japan, Tokyo, Japan) was administrated to mice under inhalation anesthesia and blood glucose concentrations were checked by a Glutest Sensor (Sanwa Kagaku Kenkyusyo CO., LTD., Nagoya, Japan) twice a week after the injection. The STZ-injected ICR mice with blood glucose levels of over 600 mg/dl were assigned as STZ-induced diabetic mice (STZ-ICR). After the single STZ injection of Pg-LPS of 3 mg/kg (LD50 = 30 mg/kg body weight; Invivogen, San Diego, California, USA) which have been confirmed to have no effect on the health condition in healthy ICR mice in our previous study was further injected with STZ-ICR just below the buccal mucosa, once a week for 4 months under inhalation anesthesia [30]. The mice were monitored for sugar, protein, and bleeding in the urine by urine reagent strips (Uriace, Terumo Corporation, Tokyo, Japan); blood which was collected from the tail vein under anesthesia was analyzed for blood urea nitrogen (BUN) and creatinine (CRE) by Kyudo Co., LTD (Tosu, Japan). When the mice showed strongly positive levels of sugar and protein in the urine by the reagent strips (Uriace, Terumo), and simultaneously showed BUN levels of above 40 mg/dl and CRE levels of above 0.7 mg/dl (Kyudo, Tosu, Japan), they were assigned as STZ and Pg-LPS-induced diabetic nephropathy mice according to our previous study [29, 30]. In summary, we performed this study using 32 ICR mice divided into four groups (*n* = 8 per group) which is the smallest number that makes it possible to achieve statistically reliable results: the healthy control ICR mice without any treatment (data not shown), control ICR mice treated with Pg-LPS (the experimental infection model, LPS-ICR), control ICR mice treated with STZ (the experimental diabetes model, STZ-ICR), and ICR mice treated with Pg-LPS and STZ (the experimental diabetic nephropathy model, LPS-STZ). At the end of the designated period of the experiments, all mice were euthanized, and tissue was collected.

### Immunohistochemistry

The immunohistochemical investigation was performed by the method described elsewhere [30]. Briefly, frozen mouse kidney tissue sections were fixed in 100% methanol and treated with primary antibodies (1 μg/ml): hamster monoclonal anti-mouse podoplanin clone 8.8.1 (#127402, BioLegend Inc., San Diego, CA, USA) to discriminate glomeruli and rabbit polyclonal anti-mouse SGLT2 (#ab85626, Abcam plc., Cambridge, UK), and rabbit polyclonal anti-type 1 collagen (#ab34710, Abcam). After the treatment sections were exposed to secondary antibodies (0.5 μg/ml): Alexa Fluor 488 and 568-conjugated goat anti-hamster and anti-rabbit IgGs (Probes Invitrogen Com., Eugene, OR, USA). Cell nuclei were counterstained with 4′, 6-diamidino-2-phenylindole (DAPI). The immunostained sections were examined by microscope digital camera systems with a CFI Plan Apo Lambda lens series and DS-Ri2/Qi2 (Nikon Corp., Tokyo, Japan). All experiments were replicated several times (5–10) with different sections. The immunostained sections used in the Figures were consecutively stained by haematoxylin and eosin (HE) to identify the immunostained regions.

### Real-time PCR

The analysis was as described elsewhere [30]. Immediately after excision 5 mm squares of tissue from mouse kidneys were ground into a paste with a scalpel on glass plates on ice and dissolved in the RLT buffer of an RNeasy kit (Qiagen, Inc., Tokyo, Japan). The total RNA extraction from the tissue was performed with a QIAshredder column and an RNeasy kit (Qiagen). When many non-specific bands were identified at the gel electrophoresis after the PCR, a DNAfree kit (Ambion, Huntingdon, UK) was used to remove contaminating genomic DNA. After the total RNA extraction, reverse transcription was performed on 30 ng of total RNA, followed by 38 cycles (annealing: 60 °C) of PCR for amplification using the Ex Taq hot start version (Takara Bio Inc., Otsu, Japan) with 50 pM of primer sets for SGLT2 mRNA (forward: CCCATCCCTCAGAAGCATCTCC; reverse: CTCATCCCACAGAACCAAAGCA) where the specificities had been confirmed by the manufacturer (Sigma-Aldrich Japan, Tokyo, Japan). The cDNA samples were also analyzed by real-time quantitative PCR to quantify the mRNA amounts. The cDNA (1 μl) was amplified in a 25-μl volume of PowerSYBR Green PCR Master Mix (Applied Biosystems, Foster City, CA, USA) in a Stratagene Mx3000P real-time PCR system (Agilent Technologies, Inc., Santa Clara, CA, USA) and the fluorescence was monitored at each cycle. Cycle parameters were 95 °C for 15 min to activate Taq followed by 40 cycles of 95 °C for 15 s, 58 °C for 1 min, and 72 °C for 1 min. Two standard curves were created for the real-time analysis from amplicons for β-actin and target genes in three serial 4-fold dilutions of cDNA. The β-actin/target gene cDNA levels in each of the sample were quantified against β-actin/target gene standard curves by allowing the Mx3000P software to accurately determine each cDNA units. Finally, the target gene cDNA amounts in each sample were normalized to β-actin cDNA. Relative amounts for the target gene expression were expressed as arbitrary units, calculated according to the following formula: relative experimental gene expression units = STZ-ICR or LPS-STZ cDNA amounts/LPS-ICR cDNA amounts. All experiments were repeated at least five times.

### Tissue ELISA

The analysis was performed by modifying the cell ELISA for tissue, and is as described elsewhere [30]. Normal mouse 5 mm square tissue of kidneys were collected after euthanasia by induction anesthesia of the mice with 2% isoflurane (1 l/min) and intraperitoneal injections with sodium pentobarbital (10 ml/kg, Nembutal, Abbott Laboratories, North Chicago, IL). Immediately after excision of the tissue, cryo-embedding in Tissue-Tek Cryomold (Sakura Finetek Japan Co., Ltd., Tokyo, Japan) was performed using liquid nitrogen. Frozen 10 μm sections cut in a cryostat on the slide glass were fixed in 100% methanol for 5 min at − 20 °C. The sections were treated with PBS blocking solution containing 0.1% goat serum for 30 min at 20 °C and then with a drop of blocking solution (0.2 ml) containing 1 μg/ml rabbit anti-mouse SGLT2 (Abcam) or rabbit anti-mouse β-actin (#8226, Abcam) for 8 h at 4 °C. After the primary antibody treatment, the sections were washed three times in PBS for 10 min and treated with a drop of blocking solution (volume 0.2 ml) containing peroxidase-conjugated second antibodies (0.1 μg/ml) for 1 h at 20 °C, and then visualized by a drop of ABTS peroxidase substrate solution (0.2 ml, SeraCare Life Sciences, Inc.) at room temperature. A 0.15 ml volume of the reaction products were added to the wells of a 96-well microplate and absorbance changes at 405 nm were measured by a microplate reader. Tissue sections treated with only a second antibody served as blanks. The produced amounts of SGLT2 protein were expressed as the mean absorbance of peroxidase metabolizing substrate of six sections and normalized to the absorbance of reaction products with rabbit anti-mouse β-actin (Abcam). Relative production amounts were expressed as arbitrary units according to the formula: STZ-ICR or LPS-STZ absorbance / LPS-ICR absorbance.

### Statistics

Animal experiments were performed with 32 mice in 4 groups (8 mice in each group) as described above. All experiments for immunohistochemistry, RT-PCR and ELISA were repeated five times. Data were expressed as the mean + SD and mean values were calculated with standard deviations. The statistical significance of the differences (*P* < 0.01) was determined by one-way ANOVA and two-tailed unpaired Student’s *t* test with STATVIEW 4.51 software (Abacus concepts, Calabasas, CA, USA). The corresponding author was fully aware of the group allocation at the different stages of the experiments. The data analysis and assessments were performed by all co-authors.

## Results

### Quantitative analysis of blood sugar levels, and SGLT mRNA and protein

**Fig. 1:**
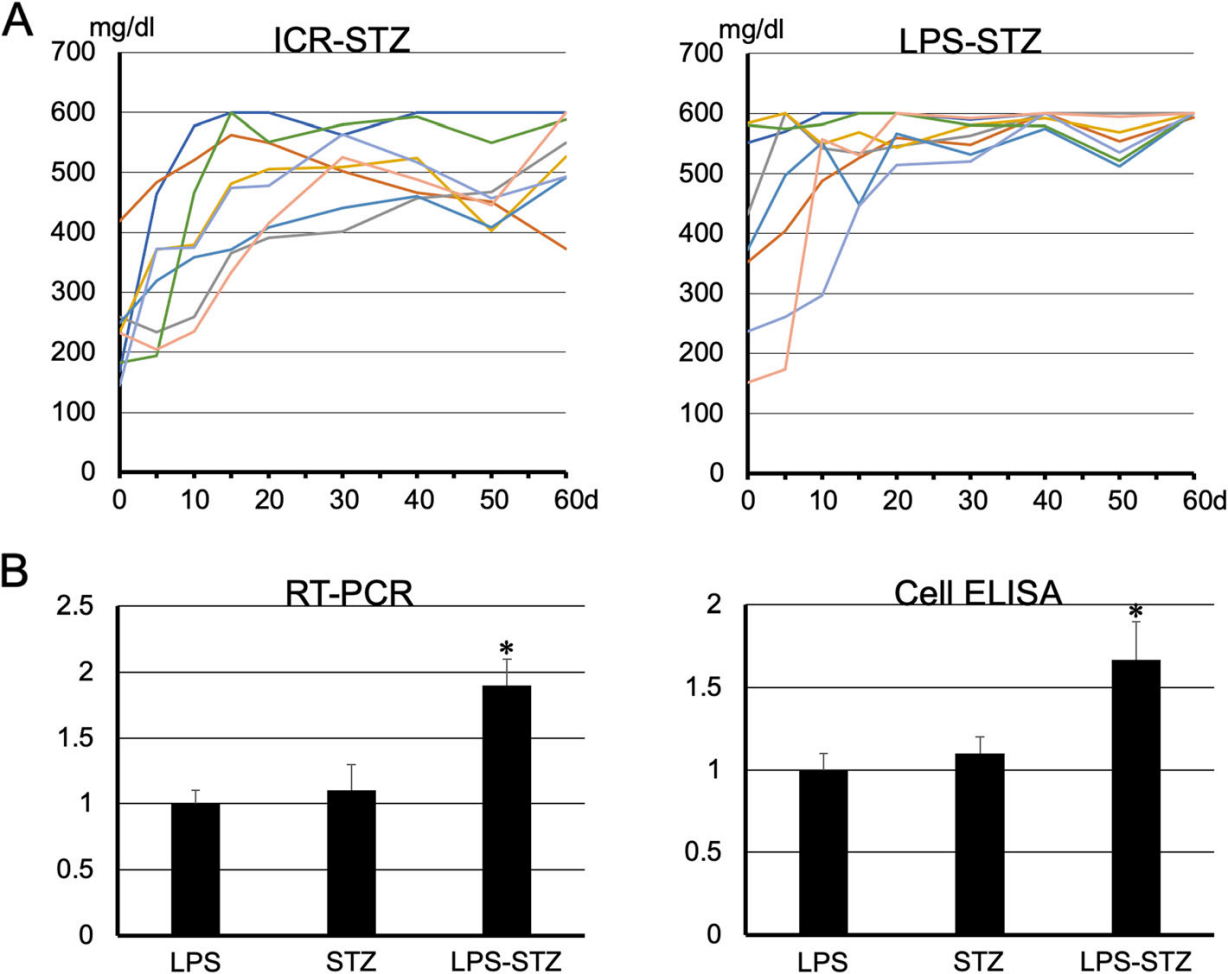
Quantitative analysis of blood sugar levels, and SGLT2 mRNA and protein of diabetic mice with Pg-LPS. (**A**) Quantitative analysis of blood sugar levels. The mean time to reach 600 mg/dl (*n* = 8) was shorter in LPS-STZ than in STZ-ICR. The increase in blood glucose levels was significantly steeper in LPS-STZ than in STZ-ICR. (**B**) RT-PCR and tissue ELISA analysis of renal SGLT2. Real time-PCR analysis shows that the amounts of gene expression of SGLT2 were significantly larger in LPS-STZ than in LPS-ICR (LPS) and STZ-ICR (STZ). There was no difference between the amounts of LPS-ICR and STZ-ICR. The cDNA amounts are normalized to the β-actin cDNA amounts. Relative gene expression amounts are expressed as arbitrary units: cDNA from STZ-ICR and LPS-STZ/cDNA from LPS. Values are mean ± SD. * Significantly different in ANOVA (*P* < 0.01). The tissue ELISA analysis of the mouse kidney sections shows that the protein production of SGLT2 was significantly larger in LPS-STZ than in LPS-ICR (LPS) and in STZ-ICR (STZ). There was no difference between the LPS and STZ amounts. The protein amounts were expressed as the mean absorbance of peroxidase metabolizing substrate of six sections and the absorbance was normalized to the absorbance with anti-β-actin. Relative produced amounts of gene/protein are expressed as arbitrary units: absorbance of STZ or LPS-STZ/absorbance of LPS. Values are mean ± SD. *Significantly different in ANOVA (*P* < 0.01)

The time to reach the blood glucose level of 600 mg/dl was shorter in the Pg-LPS-administered diabetic mice, the LPS-STZ, than in the diabetic mice without Pg-LPS, the STZ-ICR, and the slope of the rise in blood glucose levels was significantly steeper in the LPS-STZ than in the STZ-ICR specimens (Fig. 1A). The real time-PCR analysis shows that the renal gene expression of SGLT2 was significantly stronger in LPS-STZ than in the Pg-LPS-administered non-diabetic ICR mice, the LPS-ICR, and the STZ-ICR mice. There were no significant differences in the renal gene expression of SGLT2 between LPS-ICR and STZ-ICR. The tissue ELISA analysis showed that the renal production of SGLT2 protein was significantly larger in the LPS-STZ than in the LPS-ICR and STZ-ICR. There were no differences in the renal production of SGLT2 protein of the LPS-ICR and STZ-ICR (Fig. 1B).

### Immunostaining of type I collagen and SGLT2

**Fig. 2:**
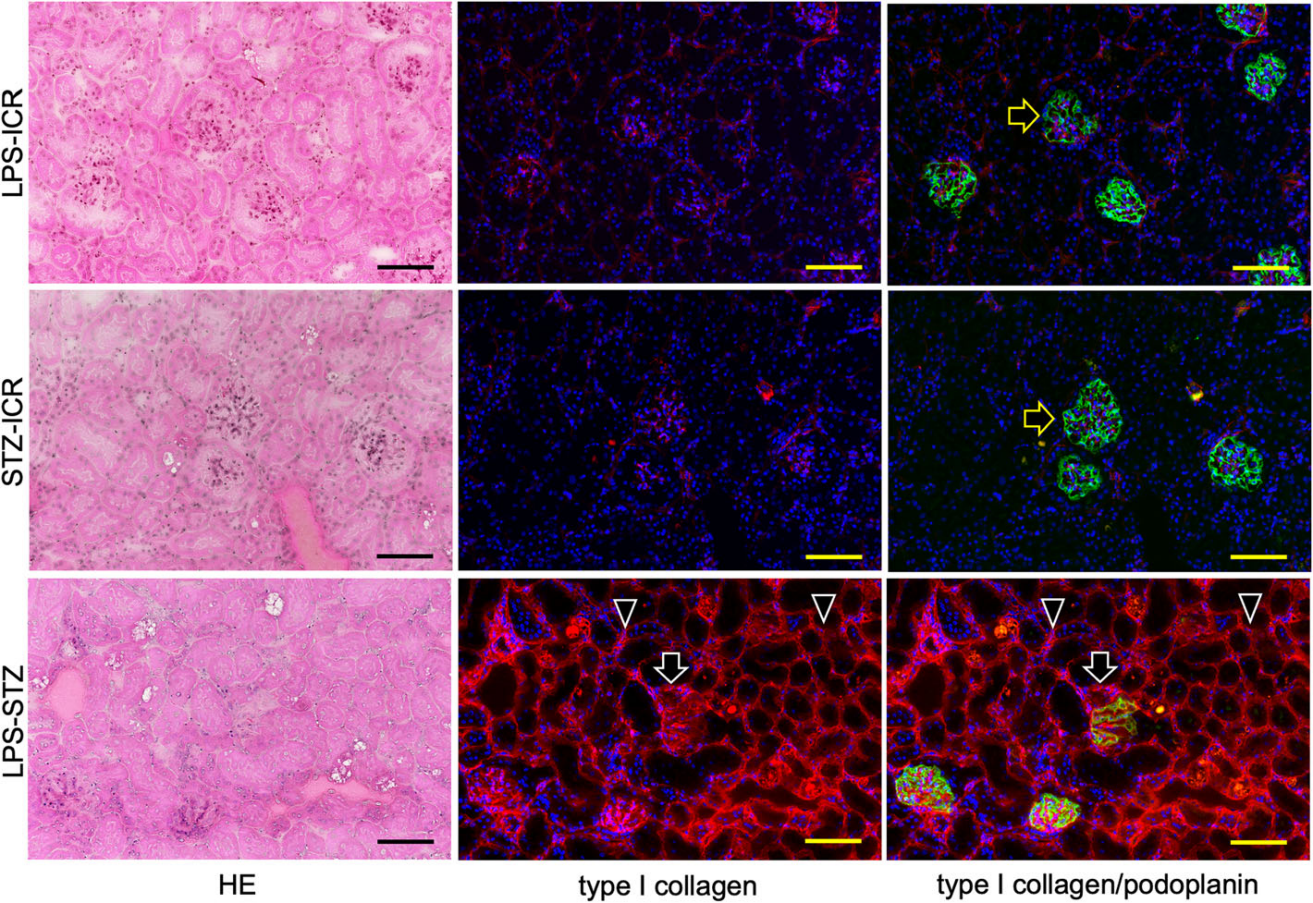
Immunostaining of type I collagen in diabetic mouse kidneys with Pg-LPS. HE staining (left column); immunostaining for type I collagen (red) (center column); and merged immunostaining for type I collagen and podoplanin (green) (right column), with DAPI staining of nuclei (blue). The glomerular epithelial cells were immunostained by anti-podoplanin to discriminate glomeruli (yellow arrows). Reaction with anti-type I collagen was weakly identified in LPS-ICR (top row) and in STZ-ICR (middle row) while the reaction was identified in the whole renal parenchyma (arrowheads) and in glomeruli (arrows) in LPS-STZ (bottom row). Bars: 100 μm

**Fig. 3:**
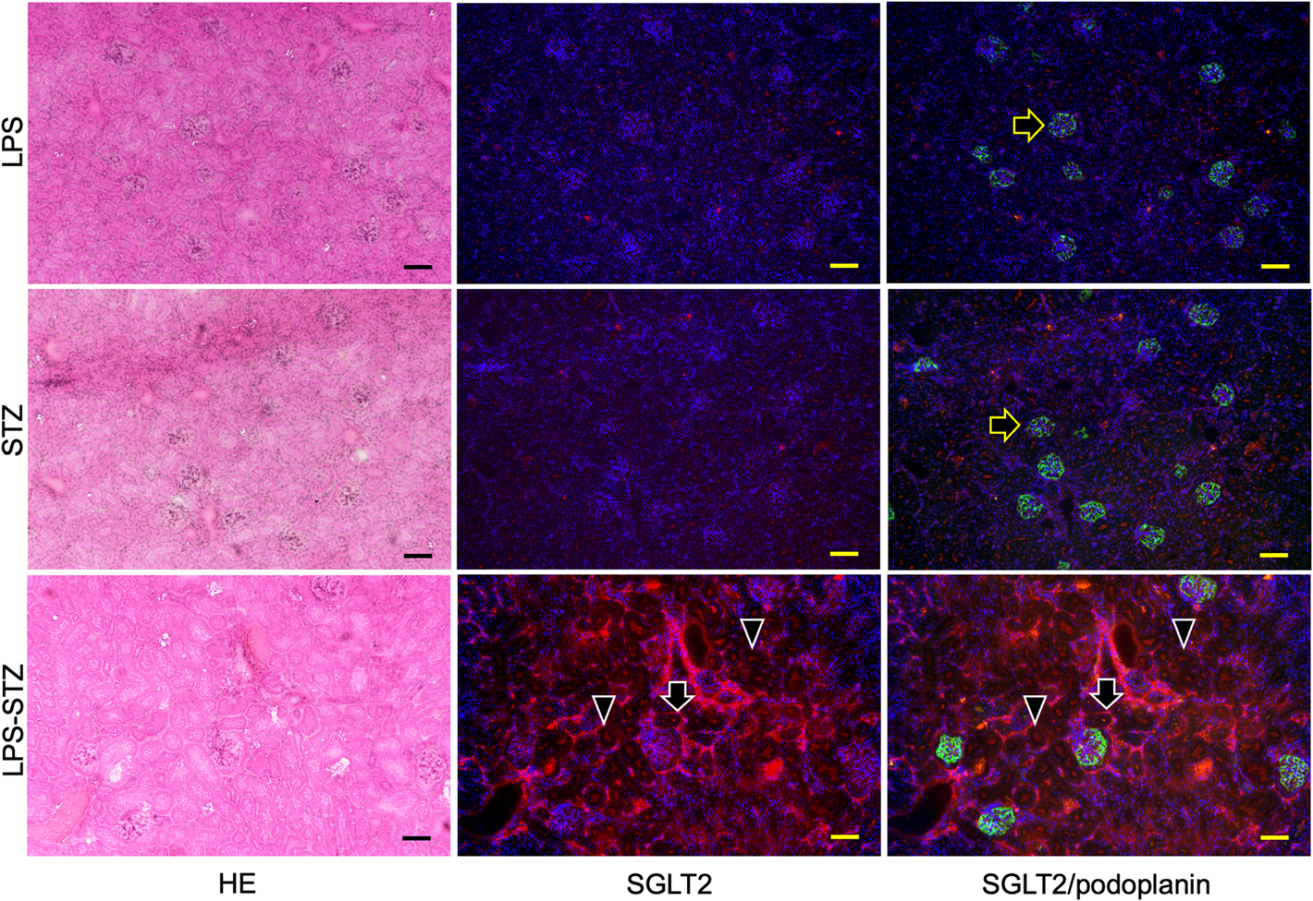
Immunostaining of SGLT2 in diabetic mouse kidneys with Pg-LPS. HE staining (left column); immunostaining for SGLT2 (red) (center column); and merged immunostaining for SGLT2 and podoplanin (green) (right column), with DAPI staining of nuclei (blue). The glomerular epithelial cells were immunostained by anti-podoplanin to be able to discriminate glomeruli (yellow arrows). Reaction with anti-SGLT2 was weakly identified in LPS-ICR (top row) and in STZ-ICR (middle row); the reaction was identified both in the proximal tubular lumen (arrowheads) and outer wall (arrows) in LPS-STZ (bottom row). Bars: 100 μm

**Fig. 4:**
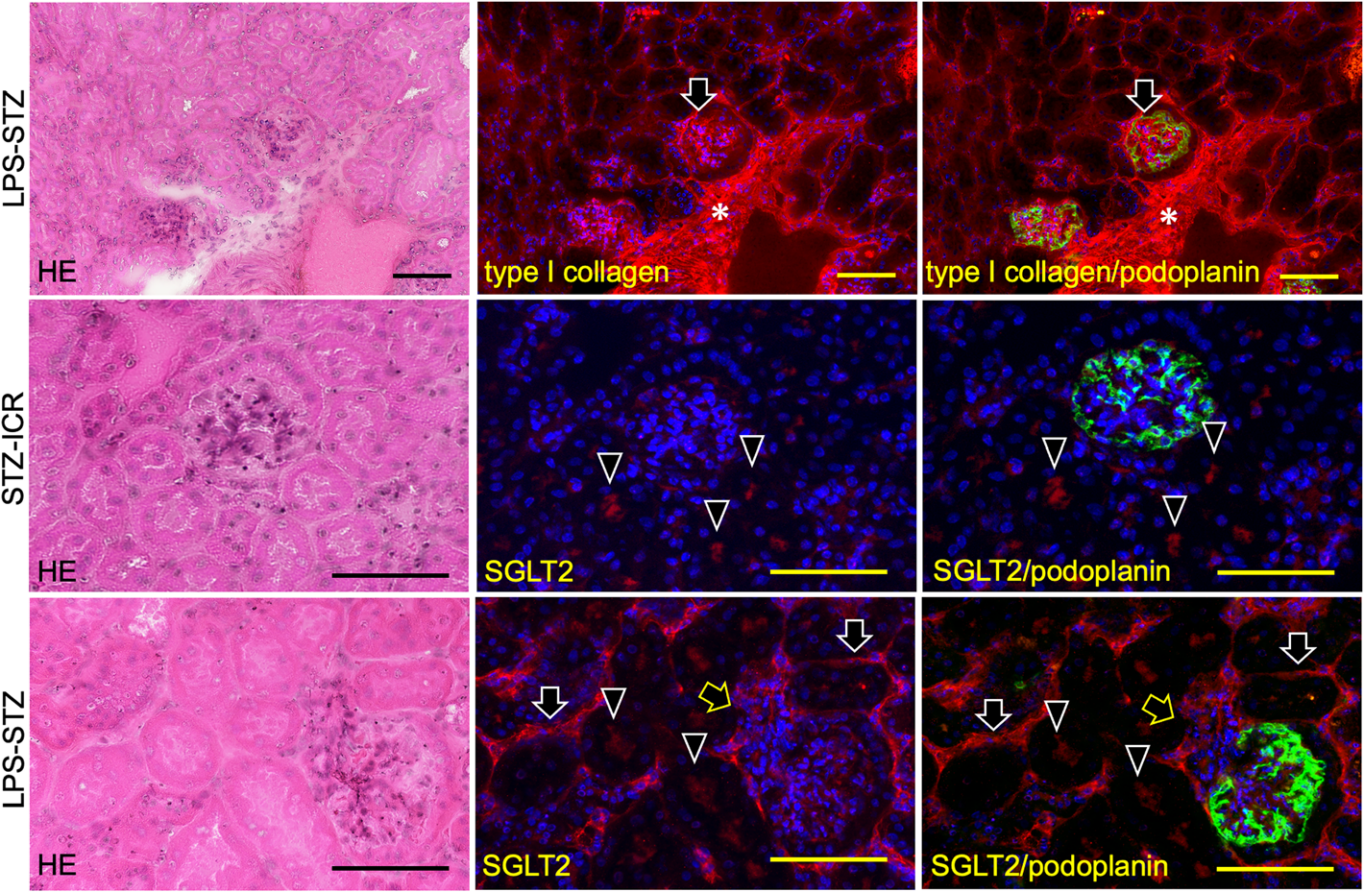
High magnification of immunostained type I collagen and SGLT2 in diabetic mouse glomeruli with Pg-LPS. HE staining (left column); immunostaining for type I collagen/SGLT2 (red) (center column); and merged immunostaining for type I collagen/SGLT2 and podoplanin (green) (right column), with DAPI staining of nuclei (blue). In LPS-STZ (top row) a strong reaction with anti-type I collagen was observed around the renal tubules, in glomeruli (arrows), and in renal columns (asterisks). In STZ-ICR (middle row) reaction to anti-SGLT2 was identified only in the proximal tubular lumen (arrowheads). In LPS-STZ (bottom row) strong reaction with anti-SGLT2 was observed at the brush border side in the renal proximal tubules (arrowheads) as well as at the outer wall of the tubules (arrows). There was a cell accumulation like leukocyte infiltration in the glomeruli (yellow arrows). Bars: 100 μm

**Fig. 5:**
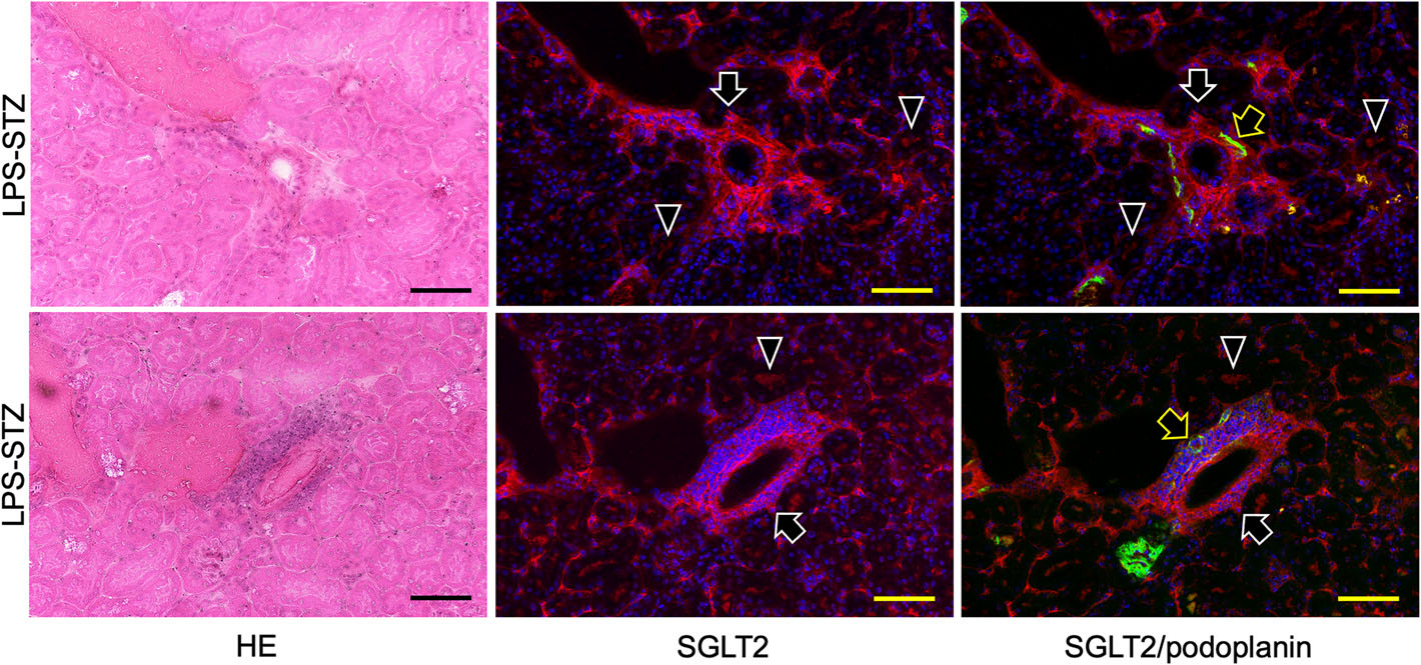
Immunostaining of SGLT2 in diabetic mouse renal vessels with Pg-LPS. HE staining (left column); immunostaining for SGLT2 (red) (center column); and merged immunostaining for SGLT2 and podoplanin (green) (right column), with DAPI staining of nuclei (blue) of LPS-STZ. In the immunostaining around a blood vessel (top row) reaction with anti-SGLT2 was observed significantly at the blood vessel side of proximal tubule outer walls (white arrows) and in the tubular lumen (arrowheads). Podoplanin-positive macrophage-like cells (yellow arrow) were observed around blood vessels. In the immunostaining of a blood vessel (bottom row) there is a leukocyte accumulation like leukocyte infiltration including podoplanin-positive macrophages (yellow arrows). A significant volume of reactions with anti-SGLT2 is observed at the blood vessel side of the proximal tubule outer walls (white arrows) and in the tubular lumen (arrowheads). Bars: 100 μm

Reaction products of anti-type I collagen were identified weakly in the ICR (not shown), LPS-ICR, and STZ-ICR specimens and strongly in the whole of the renal parenchyma and in the glomeruli of the LPS-STZ specimens (Fig. 2). There was rarely any type I collagen accumulation by administration of only Pg-LPS or STZ, but it was commonly detected in the glomerular mesangial and tubulointerstitial accumulation by Pg-LPS in the diabetic mice. Reaction products of anti-SGLT2 were weakly identified in the ICR (not shown), LPS-ICR, and STZ-ICR specimens, whereas reaction products were strongly identified in the LPS-STZ specimens in the renal proximal tubular lumen as well as at the outer wall of the tubules (Fig. 3). In the high magnification, reaction products of anti-type I collagen were observed around the renal tubules and in the glomeruli, and in the renal columns of LPS-STZ (Fig. 4). Reaction products of anti-SGLT2 were detected in only the renal proximal tubules of STZ-ICR whereas the reaction products were detected both in the renal tubules as well as at the outer wall of LPS-STZ (Fig. 4). There was leukocyte accumulation, like inflammatory leukocyte infiltration, in the glomeruli of LPS-STZ (Fig. 4). The leukocyte accumulation was observed in the juxtaglomerular apparatus of the vascular pole. In the high magnification of LPS-STZ there were strong reaction products with anti-SGLT2 detected both in the renal proximal tubular lumen and at the outer wall of the tubules, especially strongly at the tubular wall around blood vessels (Fig. 5). Leukocyte accumulation including podoplanin-positive macrophages was also observed around vessels in the renal parenchyma (Fig. 5).

## Discussion

Glomerulosclerosis is well-known as a serious complication which causes diabetic nephropathy in diabetes mellitus. We have recently reported that Pg-LPS exacerbates mouse diabetic nephropathy, and that diabetic nephropathic mice show glomerulosclerosis with the renal accumulation of type 1 collagen [28–30]. Therefore, this study used the immunostaining of type I collagen accumulation as an indicator for glomerulosclerosis. The mean time to reach 600 mg/dl was shorter in mice which had LPS-STZ with Pg-LPS administered repeatedly after a single STZ injection than in STZ-ICR mice which were only administered STZ, and the increase in blood glucose levels was significantly more rapid in LPS-STZ than in STZ-ICR (Fig. 1A). According to these data the LPS-STZ model suggests a marked glucose intolerance and it is thought that Pg-LPS induces both diabetic nephropathy as well as the diabetes. It was confirmed that the SGLT2 gene and protein expression amounts were significantly stronger in LPS-STZ than in LPS-ICR and in STZ-ICR (Fig. 1B), and there were no differences in the SGLT2 production of LPS-ICR and STZ-ICR, suggesting that *P. gingivalis* infection is the cause of the renal overexpression of SGLT2 under diabetic conditions, and that the SGLT2 overexpression does not occur only with one of diabetes or Pg-LPS. Since the glucose reabsorption by the overexpressed SGLT2 in the renal tubules is thought to be an important factor in exacerbating diabetes [31–33], these observations would allow the conclusion that periodontitis may be a critical factor in the progress of diabetes. The amounts of accumulated type I collagen in the whole of the renal parenchyma and in the glomeruli were much larger in LPS-STZ than in the kidneys of ICR (not shown), STZ-ICR, and LPS-ICR (Fig. 2). It was shown that collagen accumulation rarely occurs by only Pg-LPS or STZ administration but is strong in the glomerular mesangial and tubulointerstitial accumulation by Pg-LPS in diabetic mice (Fig. 2). In the high magnification of LPS-STZ the accumulation of type I collagen is shown to be around the renal tubules and in glomeruli, as well as in the renal columns (Fig. 4), suggesting that the kidneys of LPS-STZ became glomerulosclerotic with the high blood CRE and BUN levels described above.

In this study the expression of SGLT2 was much stronger throughout the renal parenchyma of the LPS-STZ than in kidneys of ICR (not shown), STZ-ICR, and LPS-ICR (Fig. 3). It was shown that the Pg-LPS administration induces the SGLT2 overexpression in diabetic mice but not in healthy mice (Fig. 3). In the high magnification of immunoreaction products, the SGLT2 expression was observed in the renal proximal tubular lumen of STZ-ICR (Fig. 4). Only the proximal tubular lumen was clearly stained in the diabetic mouse kidney while not only the proximal tubular lumen but also the outer walls were stained in the Pg-LPS-administered diabetic mice (Fig. 4). It has been established that the SGLT2 expression in healthy kidneys occurs only at the brush border in renal proximal tubule epithelial cells and the immunohistochemical analysis here was successfully shown in STZ-ICR (Fig. 4) [31–33]. However, in LPS-STZ, the overexpression of SGLT2 was observed not only in the proximal tubular lumen in which the secretion at the brush border is speculated to occur at the brush border side as well as at the outer wall of the tubules (Figs. 3, 4). Since there were several leukocyte accumulations resembling an inflammatory infiltration of leukocytes from glomeruli and vessels in LPS-STZ (Figs. 4, 5), it is thought that inflammatory overexpression of SGLT2 occurs in the renal proximal tubules with Pg-LPS. With the increased expression of SGLT2 in diabetic patients, SGLT2 has been suggested as an important factor in exacerbating diabetes in a production amount-dependent manner [31–33]. Considering the possibility of periodontitis as a risk factor in exacerbating nephropathy in our previous studies [28–30], it may be postulated that the overexpressed SGLT2 in the renal proximal tubular epithelial cells by the inflammatory response with *P. gingivalis* in diabetic kidneys increase the glucose reabsorption.

Commonly occurring intestinal inhabitants like *E. coli* could also be present. Such intestinal microorganisms and the LPS enter the liver via the enterohepatic circulation and are immediately sterilized, however, oral bacteria enter the systemic circulation directly without detoxification. This is the anatomical reason why head and neck infections spread to the whole body and reaction products accumulate in the kidneys [21–27]. We recently reported that the glomerular endothelium expresses TLR2/4 in diabetic mice, and that the TLR2/4-ligand *P. gingivalis* LPS is a cause of glomerulosclerosis in diabetic mice with the accumulation of type 1 collagen and inflammatory cytokines in the glomeruli [28–30]. As nephritis progresses, the SGLT2 accumulates without being metabolized due to renal dysfunction and proinflammatory cytokines like IL-6 and TNF-α induce SGLT2 expression in diabetic kidneys [39–41]. Since the expression of SGLT2 is increased in diabetic patients with persistent hyperglycemia and in patients with diabetic nephropathy, it is thought that renal glucose reabsorption by SGLT2 is a serious factor in exacerbating diabetes and that SGLT2 inhibitors are useful to prevent the development of diabetes complications [31–34]. It may be postulated that under diabetic conditions renal inflammatory events to *P. gingivalis* like the TLR recognition induce the production of inflammatory cytokines and progressively accelerate the abnormal expression of SGLT2 in glomeruli, renal proximal tubules, intertubular spaces of the renal parenchyma, and in the peritubular capillaries. Altogether this could suggest that the periodontal pathogen Pg-LPS causes the nephropathy in diabetic patients via TLR expressed by glomerular vascular endothelial cells. Further, the production of inflammatory cytokines by Pg-LPS may increase the SGLT2 expression in renal tubules and exacerbate diabetes.

## Conclusions

The results here suggest that renal inflammatory events by *P. gingivalis* like TLR recognition induce the production of inflammatory cytokines and progressively accelerate the inflammatory overexpression of SGLT2 in the renal proximal tubules under diabetic conditions. Our results suggest that periodontitis could be an exacerbating factor in both diabetic nephropathy as well as in diabetes.

## Data Availability

The datasets generated and/or analysed during the current study are not publicly available due to keep confidential but are available from the corresponding author on reasonable request.

## Abbreviations

TLR: Toll-like receptor
LPS: Lipopolysaccharide
*P. gingivalis*: *P. gingivalis*
STZ: Streptozotocin
Pg-LPS: *P. gingivalis* lipopolysaccharide
LPS-ICR: *P. gingivalis* lipopolysaccharide-administered ICR mice
STZ-ICR: Streptozotocin-induced diabetic ICR mice
LPS-STZ-ICR: Streptozotocin-induced diabetic ICR mice with *P. gingivalis* lipopolysaccharide administration
BUN: Blood urea nitrogen
CRE: Creatinine
SGLT2: Sodium-glucose cotransporter 2
HE: Haematoxylin and eosin
DAP1: 4′, 6-diamidino-2-phenylindole
